# Catheter-based ultrasound renal denervation in patients with resistant hypertension: the randomized, controlled REQUIRE trial

**DOI:** 10.1038/s41440-021-00754-7

**Published:** 2021-10-15

**Authors:** Kazuomi Kario, Yoshiaki Yokoi, Keisuke Okamura, Masahiko Fujihara, Yukako Ogoyama, Eiichiro Yamamoto, Hidenori Urata, Jin-Man Cho, Chong-Jin Kim, Seung-Hyuk Choi, Keisuke Shinohara, Yasushi Mukai, Tomokazu Ikemoto, Masato Nakamura, Shuichi Seki, Satoaki Matoba, Yoshisato Shibata, Shigeo Sugawara, Kazuhiko Yumoto, Kouichi Tamura, Fumiki Yoshihara, Satoko Nakamura, Woong Chol Kang, Taro Shibasaki, Keigo Dote, Hiroyoshi Yokoi, Akiko Matsuo, Hiroshi Fujita, Toshiyuki Takahashi, Hyun-Jae Kang, Yasushi Sakata, Kazunori Horie, Naoto Inoue, Ken-ichiro Sasaki, Takafumi Ueno, Hirofumi Tomita, Yoshihiro Morino, Yuhei Nojima, Chan Joon Kim, Tomoaki Matsumoto, Hisashi Kai, Shinsuke Nanto

**Affiliations:** 1 Division of Cardiovascular Medicine, Department of Medicine, Jichi Medical University School of Medicine, Tochigi, Japan; 2 Department of Cardiology, Kishiwada Tokushukai Hospital, Osaka, Japan; 3 Department of Cardiovascular Diseases, Fukuoka University Chikushi Hospital, Fukuoka, Japan; 4 Department of Cardiovascular Medicine, Kumamoto University Graduate School of Medical Science, Kumamoto, Japan; 5 Division of Cardiology, Department of Internal Medicine, KyungHee University Hospital at Gangdong, Seoul, South Korea; 6 Division of Cardiology, Department of Internal Medicine, CHA Gangnam Medical Center, Seoul, South Korea; 7 Division of Cardiology Heart Vascular and Stroke Institute, Department of Medicine, Samsung Medical Center, Sungkyunkwan University School of Medicine, Seoul, South Korea; 8 Department of Cardiovascular Medicine, Kyushu University Hospital, Fukuoka, Japan; 9 Division of Cardiology, Fukuoka Red Cross Hospital, Fukuoka, Japan; 10 Division of Cardiology, Kumamoto Red Cross Hospital, Kumamoto, Japan; 11 Division of Cardiovascular Medicine, Toho University Ohashi Medical Center, Tokyo, Japan; 12 Department of Cardiology, Chikamori Hospital, Kochi, Japan; 13 Department of Cardiovascular Medicine, Graduate School of Medical Science, Kyoto Prefectural University of Medicine, Kyoto, Japan; 14 Department of Cardiology, Miyazaki Medical Association Hospital, Miyazaki, Japan; 15 Department of Cardiology, Nihonkai General Hospital, Yamagata, Japan; 16 Department of Cardiology, Yokohama Rosai Hospital, Kanagawa, Japan; 17 Department of Medical Science and Cardiorenal Medicine, Yokohama City University Medical Center, Kanagawa, Japan; 18 Division of Nephrology and Hypertension, National Cerebral and Cardiovascular Center, Osaka, Japan; 19 Department of Nutritional Science for Well-being, Kansai University of Welfare Sciences, Osaka, Japan; 20 Department of Cardiology, Gil Medical Center, Gachon University College of Medicine, Incheon, South Korea; 21 Department of Cardiology, Saitama Sekishinkai Hospital, Saitama, Japan; 22 Department of Cardiology, Hiroshima City Asa Hospital, Hiroshima, Japan; 23 Cardiovascular Center, Fukuoka Sanno Hospital, Fukuoka, Japan; 24 Department of Cardiology, Japanese Red Cross Kyoto Daini Hospital, Kyoto, Japan; 25 Department of Cardiology, North Medical Center, Kyoto Prefectural University of Medicine, Kyoto, Japan; 26 Department of Cardiology, Saiseikai Central Hospital, Tokyo, Japan; 27 Department of Internal Medicine, Seoul National University Hospital and University College of Medicine, Seoul National University, Seoul, South Korea; 28 Department of Cardiovascular Medicine, Osaka University Graduate School of Medicine, Osaka, Japan; 29 Department of Cardiovascular Medicine, Sendai Kousei Hospital, Miyagi, Japan; 30 Cardiovascular Center, Tokyo Kamata Hospital, Tokyo, Japan; 31 Division of Cardiovascular Medicine, Department of Internal Medicine, Kurume University School of Medicine, Fukuoka, Japan; 32 Division of Cardiology, Fukuoka Kinen Hospital, Fukuoka, Japan; 33 Department of Cardiology, Hirosaki University Graduate School of Medicine, Aomori, Japan; 34 Division of Cardiology, Department of Internal Medicine, Iwate Medical University, Iwate, Japan; 35 Department of Cardiovascular Medicine, Nishinomiya Municipal Central Hospital, Hyogo, Japan; 36 Division of Cardiology, Department of Internal Medicine, Uijeongbu St Mary’s Hospital, College of Medicine, The Catholic University of Korea, Seoul, South Korea; 37 Department of Cardiology, Oji General Hospital, Hokkaido, Japan; 38 Department of Cardiology, Kurume University Medical Center, Fukuoka, Japan

**Keywords:** Ambulatory blood pressure, Hypertension, Renal denervation, Sham procedure, Systolic blood pressure.

## Abstract

**Abstract:**

Renal denervation is a promising new non-pharmacological treatment for resistant hypertension. However, there is a lack of data from Asian patients. The REQUIRE trial investigated the blood pressure-lowering efficacy of renal denervation in treated patients with resistant hypertension from Japan and South Korea. Adults with resistant hypertension (seated office blood pressure ≥150/90 mmHg and 24-hour ambulatory systolic blood pressure ≥140 mmHg) with suitable renal artery anatomy were randomized to ultrasound renal denervation or a sham procedure. The primary endpoint was change from baseline in 24-hour ambulatory systolic blood pressure at 3 months. A total of 143 patients were included (72 renal denervation, 71 sham control). Reduction from baseline in 24-hour ambulatory systolic blood pressure at 3 months was not significantly different between the renal denervation (−6.6 mmHg) and sham control (−6.5 mmHg) groups (difference: −0.1, 95% confidence interval −5.5, 5.3; *p* = 0.971). Reductions from baseline in home and office systolic blood pressure (differences: –1.8 mmHg [*p* = 0.488] and −2.0 mmHg [*p* = 0.511], respectively), and medication load, did not differ significantly between the two groups. The procedure-/device-related major adverse events was not seen. This study did not show a significant difference in ambulatory blood pressure reductions between renal denervation and a sham procedure in treated patients with resistant hypertension. Although blood pressure reduction after renal denervation was similar to other sham-controlled studies, the sham group in this study showed much greater reduction. This unexpected blood pressure reduction in the sham control group highlights study design issues that will be addressed in a new trial.

**Clinical trial registration:**

NCT02918305 (http://www.clinicaltrials.gov).

## Introduction

Hypertension is a common problem, affecting >1.1 billion people worldwide [1, 2]. Unfortunately, fewer than one in five treated patients with hypertension have their blood pressure (BP) under control [2]. The increasing number of people with uncontrolled BP despite a greater number of therapeutic options has been described as the “hypertension paradox” [3]. Achieving BP control is essential because patients with hypertension who have uncontrolled BP have significantly higher rates of all-cause, cardiovascular, heart disease and cerebrovascular disease mortality compared to normotensive individuals, whereas mortality risk in patients with well-controlled BP does not differ from that in normotensive individuals [4].

There are a number of potential factors that contribute to the suboptimal control of hypertension, including medication non-adherence and prescribing inertia [5, 6]. This highlights the limitations of purely pharmacological approaches for the effective management of hypertension.

Over the last decade, catheter-based renal denervation has emerged as a potential treatment option for patients with resistant hypertension. Proof-of-concept trials reported dramatic BP-lowering effects in patients treated with radiofrequency catheter-based renal denervation [7, 8]. However, enthusiasm was tempered by the neutral findings of the randomized, sham-controlled SYMPLICITY HTN-3 trial [9], although several confounding variables were identified that might explain the study results [10]. Nevertheless, trials with second-generation radiofrequency- and ultrasound-based renal denervation devices have reported promising results in proof-of-concept [11, 12] and adequately powered trials [13–16].

The SYMPICITY HTN-JAPAN trial [17] was stopped early when SYMPLICITY HTN-3 failed to meet its primary efficacy endpoint [9]. Therefore, there is a limited amount of data on the use of renal denervation in patients of Asian ethnicity [18], who have a different hypertension phenotype and hypertension-related cardiovascular risk compared with Caucasians [19–25]. The sham-controlled REnal denervation on Quality of 24-hr BP control by Ultrasound In REsistant hypertension (REQUIRE) trial was designed to assess the BP-lowering efficacy of renal denervation in treated patients with resistant hypertension from Japan and South Korea [26].

## Methods

### Study design and oversight

The REQUIRE trial was a multicenter (*n* = 72), randomized, single-blind, sham-controlled trial that enrolled patients from Japan and South Korea (see online data supplement) between January 12, 2017 and March 31, 2020. The trial received ethical approval from the institutional review boards at each study site, and all patients provided written informed consent prior to enrollment. The trial was conducted in accordance with Good Clinical Practice guidelines and the provisions of the Declaration of Helsinki.

### Study participants

Full details of patient inclusion and exclusion criteria have been reported previously [26]. Briefly, eligible patients were aged 20–75 years and had resistant hypertension (average seated office BP ≥ 150/90 mmHg) despite treatment with a stable regimen including maximum tolerated dosages of at least three antihypertensive medications from different classes (including a diuretic) and 24-hour ambulatory systolic BP (SBP) of ≥140 mmHg during a screening period of ~4–8 weeks prior to the procedure. Renal artery anatomy eligibility was determined using computed tomography or magnetic resonance angiogram at the end of the screening period, then confirmed by renal artery angiography at the time of procedure. Patients with unsuitable renal artery anatomy were excluded, as were those with chronic kidney disease (estimated glomerular filtration rate <40 mL/min/1.73 m^2^), secondary hypertension (although patients with sleep apnea were eligible), inadequately controlled diabetes mellitus, inflammatory bowel disease, history of severe cardiovascular event, or other chronic conditions.

### Randomization and blinding

Patients were randomized in a 1:1 ratio to undergo renal denervation using the Paradise ^TM^ Renal Denervation System (ReCor Medical Inc., Palo Alto, CA, USA) or to a sham procedure (renal angiogram only). Randomization was performed using a web-based randomization tool and was stratified by country (South Korea or Japan), study site, and baseline 24-hour ambulatory SBP (140 to <160 mmHg or ≥160 mmHg). Subjects remained blinded to treatment allocation until 6 months after the procedure. All physicians and study coordinators, including those who interacted with patients, were aware of treatment allocation, but BP assessments were performed by study personnel who were unaware of treatment allocation.

### Interventions

The catheter-based Paradise^TM^ Renal Denervation System thermally ablates the renal sympathetic nerves by delivering circumferential ultrasound energy. The system includes a single-use 6-French catheter and an automated, portable, customized generator. Full details are provided in the study methods publication [26].

Subjects in the study underwent renal denervation using minimum of two 7-second ultrasound sonications delivered bilaterally to the main renal artery; at least one sonication was delivered within accessory arteries of ≥4 mm and ≤8 mm in diameter. The sham control group underwent a renal angiogram without denervation and stayed in the catheterization laboratory with the sheath inserted for ≥20 min.

Standard-of-care antihypertensive medication was to remain unchanged up to the 3-month follow-up data collection.

### Outcomes

The primary endpoint was the between-group difference in change in 24-hour ambulatory SBP from baseline at 3 months. Secondary endpoints were change in daytime and nighttime ambulatory SBP from baseline at 3 months, change in 24-hour, daytime and nighttime ambulatory diastolic BP (DBP) from baseline at 3 months, and change in seated office SBP and DBP from baseline at 3 months. Other prespecified observational endpoints include change in home SBP and DBP.

### Assessments

All BP measurements were determined according to relevant Japanese guidelines at the time the study was designed [27, 28]. Office BP was determined at each study visit, including screening, baseline, discharge and at months 1, 2, and 3. Office BP measurements were performed using a validated automated device (OMRON HEM-907; Omron Healthcare Corp., Kyoto, Japan) on the same arm with the patient in a seated position. The value at each visit was determined from the average of three consecutive stable values.

Ambulatory BP was measured at baseline, and month 3 after renal denervation, using a validated device (TM-243 series; A & D Co., Tokyo, Japan). During ambulatory BP monitoring, BP was measured at 30-min intervals. Measurements were taken every 30 min for 25-hours and mean 24-hour BP was calculated as the average of all successful readings after excluding the first 1-hour of measurements. Participants recorded the times that they fell asleep and woke up in a diary. They were instructed to rest or sleep during the nighttime and to maintain their usual daytime activities. Nighttime BP readings were those recorded from the time of falling asleep to the time of waking up; all other values were defined as daytime readings.

Home BP was measured for 7 days before study visits at baseline, and months 1, 2, and 3. Home BP measurements were performed with a validated device (OMRON HEM-7080IC; Omron Healthcare Corp., Kyoto, Japan). Four measurements were taken each day (two times before breakfast and two times before bedtime). The first day of the measurement was excluded, and data were considered valid and averaged if all four measurements were available for ≥3 days. Patients were instructed to store BP values in their home BP monitoring device for download at the next study visit.

The number of antihypertensive medications used was determined at all study visits, and the antihypertensive load index (sum of daily dose/maximum daily dose for each antihypertensive drug) [29] was calculated at baseline and 3-month follow-up.

Safety data, including all adverse events regardless of their relationship to the study procedure, were collected for up to 12 months after the procedure (see previous publication for full details) [26]. In brief, the following 30-day safety events were determined: any renal artery complication requiring intervention (e.g., dissection and perforation); complications in the inguinal or femoral region, iliac artery, or abdominal aorta requiring intervention; significant embolic events resulting in end-organ damage; procedure-related pain lasting for >2 days; acute renal failure; bleeding requiring blood transfusion or surgery; and pseudo aneurysm.

### Sample size calculation

Assuming that the reduction in 24-hour ambulatory SBP would be 6 mmHg greater in the renal denervation group than in the sham control group (standard deviation, 12 mmHg) [17, 30–32], it was calculated that the number of patients required to detect a difference between the renal denervation and the sham control groups with 80% power and a two-sided significance level of 5% was 128 (64 per group). Allowing for a 10% dropout rate over the first 3 months after the procedure, the target sample size was 140 (70 per group).

### Statistical analysis

Data are presented as mean values with standard deviation, and number of patients with percentages. Efficacy analyses for BP values were conducted in the full analysis set (including all patients with ≥75% valid data for 24-hour ambulatory BP from baseline to 3 months), and safety was determined in the safety analysis set which included all randomized patients (excluded for patients without ablate the renal sympathetic nerves in the renal denervation group).

Analysis of covariance (ANCOVA) modeling with baseline values as covariates was used to compare the changes of least square mean in 24-hour BP, daytime BP, nighttime BP, home BP, and office BP from baseline at 3 months. ANCOVA modeling included the randomized study group, time point (1, 2, and 3 months), interaction between the study group and time points as fixed effects, and baseline values as covariates.

All statistical analyses were pre-specified before the final analysis, and were performed with SAS system, v9.4 (SAS Institute, Cary, NC, USA). Two-sided *p* < 0.05 were defined as statistically significant.

## Results

### Study population

**Fig. 1:**
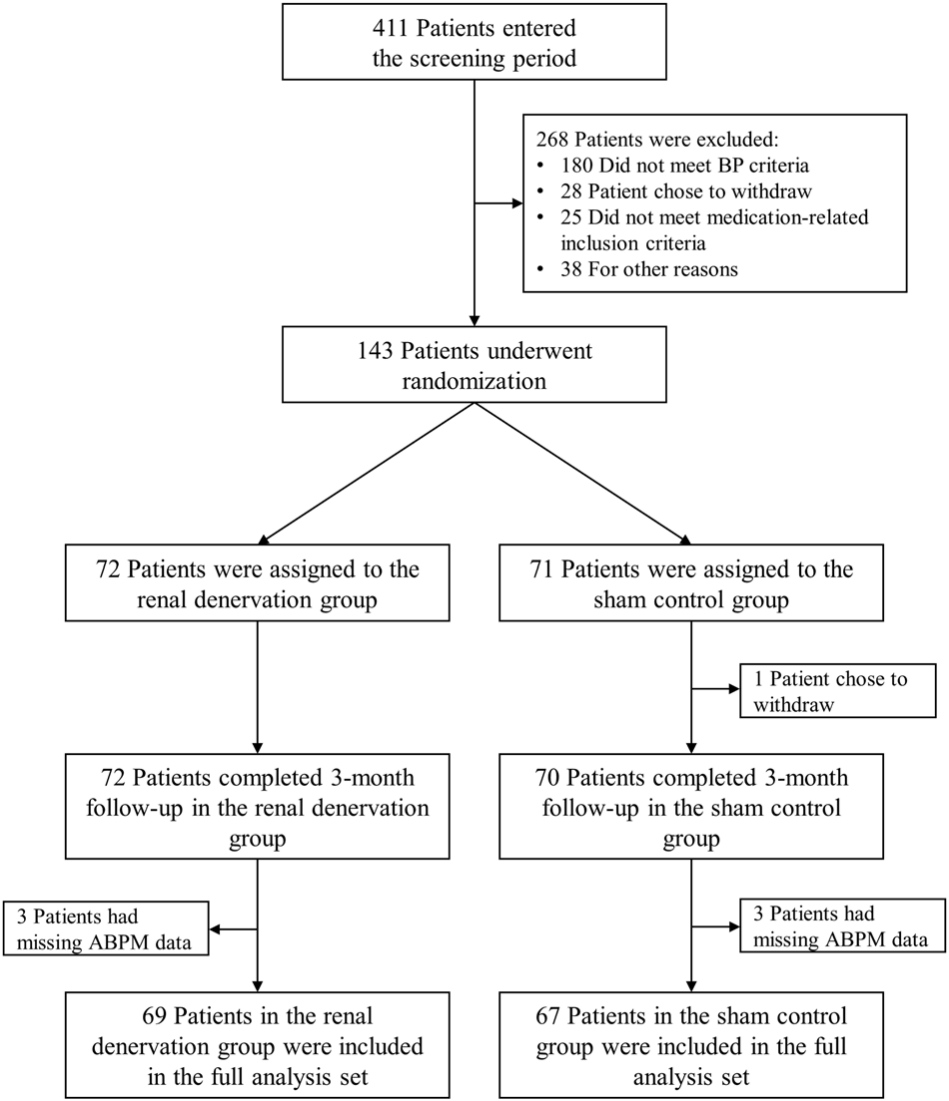
Flow chart of study participants. Randomization, procedures and follow-up (data cut-off September 30, 2020)

A total of 411 patients entered the screening period, of whom 143 met all eligibility criteria and were included in the study (72 in the renal denervation group and 71 in the sham control group); all but one patient completed the three-month follow-up (one patient in the sham control group withdrew from the study) (Fig. 1, Table 1).

**Table 1:** Demographic and clinical characteristics of the patients at baseline

Variables	Renal denervation (*n* = 69)	Sham control (*n* = 67)
Age, year	50.7 ± 11.4	55.6 ± 12.1
Female, *n* (%)	21 (30.4)	14 (20.9)
Body mass index, kg/m^2^	29.5 ± 5.5	28.4 ± 4.5
eGFR, mL/min per 1.73m^2^	74.2 ± 16.2	69.6 ± 17.1
eGFR <60 mL/min per 1.73m^2^, *n* (%)	15 (21.7)	18 (26.9)
Comorbidities, *n* (%)		
Cardiovascular disease	9 (13.0)	9 (13.4)
Diabetes mellitus	18 (26.1)	20 (29.9)
Dyslipidemia	39 (56.5)	40 (59.7)
Peripheral arterial disease	1 (1.4)	2 (3.0)
Cerebrovascular disease	0 (0.0)	5 (7.5)
Sleep apnea syndrome	11 (15.9)	8 (11.9)
Aortic dissection	1 (1.4)	0 (0.0)
Office blood pressure, mmHg		
Systolic	157.6 ± 19.5 (*n* = 69)	160.4 ± 14.9 (*n* = 66)
Diastolic	97.7 ± 16.6 (*n* = 69)	95.3 ± 14.2 (*n* = 66)
Office pulse rate, beats/min	75.3 ± 10.8 (*n* = 69)	71.5 ± 12.8 (*n* = 66)
Ambulatory blood pressure, mmHg		
24-hour systolic	161.9 ± 13.4 (*n* = 69)	161.5 ± 13.1 (*n* = 67)
24-hour diastolic	94.9 ± 9.3 (*n* = 69)	92.7 ± 9.4 (*n* = 67)
Daytime systolic	166.7 ± 13.1 (*n* = 64)	167.3 ± 13.8 (*n* = 66)
Daytime diastolic	97.9 ± 9.7 (*n* = 64)	96.2 ± 9.6 (*n* = 66)
Nighttime systolic	149.9 ± 18.9 (*n* = 69)	150.1 ± 18.1 (*n* = 67)
Nighttime diastolic	86.7 ± 11.0 (*n* = 69)	85.5 ± 11.2 (*n* = 67)
Home blood pressure, mmHg		
Systolic	163.5 ± 18.7 (*n* = 63)	163.3 ± 15.4 (*n* = 62)
Diastolic	98.0 ± 13.7 (*n* = 63)	93.4 ± 13.9 (*n* = 62)
Number of antihypertensive drugs, *n* (%)	4.1 ± 1.6	3.9 ± 1.1
3	32 (46.4)	29 (43.3)
4	20 (29.0)	23 (34.3)
≥5	17 (24.6)	15 (22.4)
Antihypertensive drug classes, *n* (%)		
RAS blocker	68 (98.6)	66 (98.5)
Calcium channel blocker	63 (91.3)	59 (88.1)
Diuretic	64 (92.8)	63 (94.0)
MR blocker	17 (24.6)	10 (14.9)
α-blocker	14 (20.3)	12 (17.9)
β-blocker	24 (34.8)	25 (37.3)
α-/β-blocker	15 (21.7)	17 (25.4)
Centrally acting agent	6 (8.7)	3 (4.5)
Vasodilator	0 (0.0)	0 (0.0)

Values are mean ± standard deviation, or number of patients (%)

*eGFR* estimated glomerular filtration rate; *MR* mineralocorticoid receptor; *RAS* renin angiotensin system

### Study intervention and follow-up

Procedure time (86.7 vs 40.6 min), x-ray fluoroscopy time (23.6 vs 5.2 min) and contrast volume (147.8 vs 54.1 mL) were higher in the renal denervation versus sham control group. Overall, 71/72 (98.6%) renal denervation patients had at least two sonications in each renal artery (Supplementary Table 1).

Valid ambulatory BP monitoring data at 3 months were available for 69 patients in the renal denervation group and 67 patients in the sham control group (full analysis set; Fig. 1).

### Twenty-four-hour ambulatory blood pressure

**Fig. 2:**
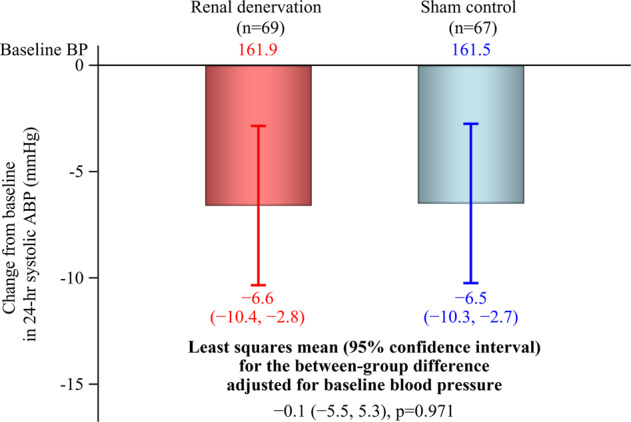
Change from baseline in 24-hour ambulatory systolic blood pressure at 3 months after the procedure. ABP, ambulatory blood pressure. Bars and error bars show least squares mean ± 95% confidence interval. Numbers below the error bars refer to least squares mean ± 95% confidence interval

The reduction from baseline in 24-hour ambulatory SBP at 3 months (primary endpoint) was not significantly different between two groups (between-group difference at 3 months: −0.1, 95% confidence interval −5.5, 5.3; *p* = 0.971) (Fig. 2). The lack of any statistically significant difference between the renal denervation and sham control groups in 24-hour ambulatory SBP was consistent across patient subgroups based on age, sex, country, and baseline values of 24-hour ambulatory, seated office and home SBP (Supplementary Fig. 1).

Approximately half of the patients in both groups showed a decrease in 24-hour ambulatory SBP at 3 months after the procedure (Supplementary Fig. 2). The proportion of patients with a ≥5 mmHg decrease in 24-hour ambulatory SBP was 53.6% in the renal denervation group and 49.3% in the sham control group.

There were no significant between-group differences in daytime and nighttime ambulatory BP between the two groups (Supplementary Table 2). Furthermore, 24-hour ambulatory BP profiles were similar before and after the procedure in both groups (Supplementary Fig. 3).

### Home and office blood pressure

**Fig. 3:**
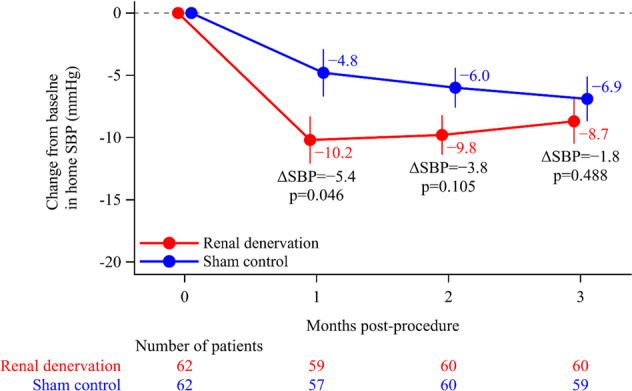
Change in home systolic blood pressure (SBP) over time after the procedure. Dots and error bars show least squares mean ± standard errors

At one-month post-procedure, home SBP decreased to a significantly greater extent from baseline in the renal denervation versus sham control group, but between-group differences in the change from baseline were no longer statistically significant at months two and three (Fig. 3). There were also no statistically significant differences in office BP between the renal denervation and control groups at the 3-month follow-up (Supplementary Table 2).

### Medication

The number of antihypertensive medications and the antihypertensive load index was similar in both groups throughout the study (Supplementary Table 3). Antihypertensive medications were changed in fifteen patients (nine in the renal denervation group [up-titrated in 3, down-titrated in 1, and change of drugs in 5] and six in the sham control group [up-titrated in 2, down-titrated in 2, and change of drugs in 2]). In a post-hoc analysis, differences in the change in BP between the renal denervation and sham control groups in patients without a change in antihypertensive therapy were consistent with those of the main analysis (Supplementary Table 4). At both one and two months post-procedure, patients without any change in antihypertensive drugs showed a significantly greater reduction from baseline in home SBP after treatment with renal denervation compared with a sham procedure (between-group difference of −7.3 mmHg [*p* = 0.004] and −4.4 mmHg [*p* = 0.050], respectively), but between-group differences in the change from baseline were no longer statistically significant at the 3-month follow-up (Supplementary Fig. 4).

### Post-hoc analysis excluding patients with hyperaldosteronism

When being treated with three or more antihypertensive drugs including renin-angiotensin-aldosterone inhibitors, eighteen patients in the renal denervation group and 26 patients in the sham control group showed hyperaldosteronism (defined based on an aldosterone/renin ratio >200 [calculated as aldosterone concentration in pg/mL/plasma renin activity in ng/mL/h] and aldosterone concentration >120 pg/mL). In a post-hoc analysis excluding these 44 patients, the reduction in 24-hour ambulatory SBP from baseline at 3 months was −7.6 mmHg in the renal denervation group and −4.2 mmHg in the sham control group (between-group difference −3.3 mmHg, not significant), and the reduction in home SBP from baseline to 1 month was −12.1 mmHg in the renal denervation group and −3.6 mmHg in the sham control group (between-group difference −8.5 mmHg, *p* = 0.012).

### Safety

The procedural success rate was high (98.6%). The procedure-/device-related major adverse events was not seen. The most common specific clinical events were procedure-related pain lasting for >2 days (e.g., back pain, puncture site pain, etc.), which occurred in six patients in each group (Table 2). Vasospastic angina and a puncture site hemorrhage occurred in one patient each during the renal denervation procedure (Table 3).

**Table 2:** Specific clinical events within 30 days post-procedure

	Renal denervation (*n* = 72)	Sham control (*n* = 71)
Vasospasm of renal artery treated with medication^a^	4 (5.6%)	0
Any renal artery complication requiring intervention	0	0
Complication of iliac artery or abdominal aorta requiring intervention	0	0
Complication at femoral puncture site^b^	4 (5.6%)	3 (4.2%)
Significant embolic events resulting in end organ damage	0	0
Procedure-related pain lasting for >2 days	6 (8.3%)	6 (8.5%)
Acute renal failure	0	0
Bleeding requiring blood transfusion or surgery	0	0
Pseudo aneurysm	0	0

^a^Required intra-arterial injection of nitrates. All events resolved quickly during the procedure with this treatment.

^b^Pain (*n* = 4), skin injury (*n* = 1), hematoma (*n* = 2); one hematoma in the renal denervation group required a balloon catheter.

**Table 3:** Serious procedure-/device-related adverse events within 3 months

	Renal denervation (*n* = 72)	Sham control (*n* = 71)
Vasospastic angina (Prinzmetal angina)	1 (1.4%)	0
Puncture site hemorrhage	1 (1.4%)	0
Pyrexia	0	1 (1.4%)
Cellulitis	1 (1.4%)	0
Blood pressure decreased	1 (1.4%)	0
Blood pressure increased	1 (1.4%)	0
Postural dizziness	1 (1.4%)	0

## Discussion

**Fig. 4:**
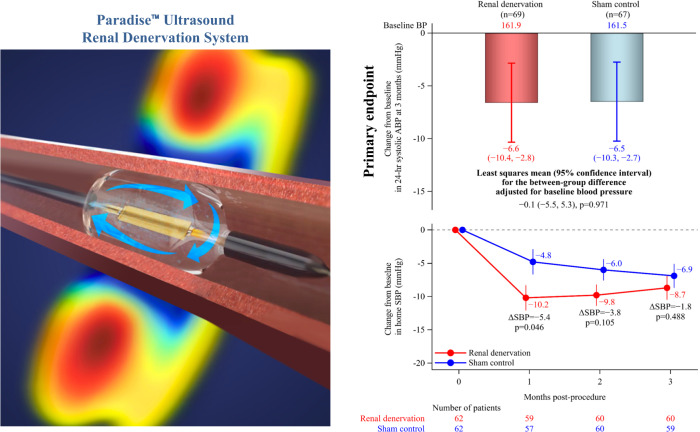
Graphical Abstract: Although BP decreased significantly from baseline in the ultrasound renal denervation group, this trial had a neutral result because there was a similar reduction in BP in the sham control group

The REQUIRE trial is the first trial of ultrasound renal denervation in Asian patients with hypertension receiving antihypertensive therapy. The study findings were neutral for the primary endpoint, with similar reductions in 24-hour ambulatory SBP in the renal denervation and sham control groups (Fig. 4).

The field of renal denervation underwent a major reappraisal following the SYMPLICITY HTN-3 results [9], which showed no difference in BP outcomes between patients treated with point-by-point radiofrequency renal denervation versus a sham control. Global committees of experts recommended a number of important trial design changes including: (1) standardization of the renal denervation procedure; (2) measurement of ambulatory BP as a primary outcome; (3) standardization of medications; and (4) measurement of medication adherence [33]. The recently published RADIANCE-HTN TRIO (a sham-controlled randomized study in patients with hypertension resistant to a guideline-approved single-pill, triple combination therapy) trial implemented all of these recommendations and met its primary endpoint in a resistant hypertension population [16]. Based on the available body of data from sham-controlled trials for the efficacy and safety of renal denervation, a recent European Society of Hypertension position paper [34] describes this procedure as an evidence-based option for the treatment of hypertension, in addition to lifestyle modifications and pharmacological antihypertensive therapy.

The results presented here are particularly interesting considering the findings of the SYMPLICITY HTN-3 [9] and RADIANCE-HTN TRIO [16] studies. Like RADIANCE-HTN TRIO, the REQUIRE trial utilized a newer device that may allow for a more repeatable procedure and had ambulatory BP as the primary endpoint. However, unlike RADIANCE-HTN TRIO [16], REQUIRE did not standardize medications and objectively measure medication adherence. In addition, blinding was not complete and treating physicians and coordinators following study subjects might have been aware of the initial treatment assignment. In the setting of resistant hypertension, medication adherence and variability may pose an important challenge to trial design and cause confounding of results, perhaps contributing to the neutral results observed in SYMPLICITY HTN-3 [9] and REQUIRE.

Although the between-group difference in the primary endpoint in the REQUIRE trial was not statistically significant, the absolute magnitude of the reduction from baseline in 24-hour ambulatory SBP in the renal denervation group (−6.6 mmHg) was of a similar magnitude to decreases in this parameter in eight previous sham-controlled clinical trials [9, 11–13, 16, 35–37], including those using the same ultrasound renal denervation device [13, 14, 16]. The 24-hour ambulatory BP reduction in the RADIANCE-HTN SOLO trial (a sham-controlled randomized study using the same renal denervation device as the current trial on hypertensive patients without medication) was −7.0 mmHg [13], and the corresponding reduction in the RADIANCE-HTN TRIO trial was −8.5 mmHg [16]. Thus, the magnitude of 24-hour BP reductions in the renal denervation groups from the three prospectively-powered sham-controlled trials using ultrasound renal denervation (REQUIRE, RADIANCE-HTN SOLO, and RADIANCE-HTN TRIO) were comparable.

The key difference between the current REQUIRE trial and all previous studies was that the reduction from baseline in 24-hour ambulatory SBP in the sham control group was much greater. In the RADIANCE-HTN SOLO and TRIO studies, the magnitude of reductions in 24-hr ambulatory SBP from baseline in the sham control group were −3.1 mmHg and −2.9 mmHg, respectively, whereas the reduction from baseline in the control group in this study was −6.5 mmHg. Furthermore, sham control groups in most other trials showed a change in 24-hour ambulatory SBP of −0.05 to −3.5 mmHg [9, 11–13, 35, 36].

In trying to understand the comparatively large reduction in BP in the sham control group in the current study, we wondered whether patient selection might have contributed to this. It is possible that a significant number of unstable patients with uncontrolled hypertension and poor drug adherence were enrolled in the study. These patients with poor drug adherence at baseline may have improved their medication taking after the procedure (possibly as a result of increased trial-related healthcare interactions), which would contribute to reducing BP. For example, 10.1% of patients in the renal denervation group and 7.5% of those in the control group recorded at least a 30% reduction in 24-hour ambulatory SBP by the 3-month follow-up. Optimization of medical therapy along with improved adherence could have been responsible for at least part of these substantial BP reductions. Differing degrees of BP optimization secondary to improving adherence between groups could have occurred as a result of patient unblinding either by the use of home BP monitoring or by inadvertent communications by unblinded staff. Unfortunately, we did not collect blinding index information, nor do we have medication metabolite adherence data to truly know the underlying cause.

Less stable BP prior to renal denervation, resulting in better control during the study, was suggested as a contributor to the large placebo effect in a previous renal denervation trial [37] and may also have played a role in our study. In the RADIANCE-HTN TRIO trial, patients were being treated with a fixed-dose, single-pill, 3-drug combination prior to randomization. The single-pill regimen was associated with good adherence to therapy (≈80% through the trial as measured by urine chemical adherence testing using liquid chromatography-mass spectrometry) and did minimize pre-study differences in adherence and any heterogeneous effects of different drug treatments between the renal denervation and sham control groups. In contrast, in the REQUIRE trial, patients were being treated with any combination of ≥3 antihypertensive agents before randomization, without any standardization of regimen or requirement for a fixed drug combination. Use of regimens including multiple single antihypertensive drugs would likely reduce adherence compared with the fixed combination used in RADIANCE-HTN TRIO, because regimens that include fewer pills, such as single-pill combinations, are consistently associated with better adherence and higher rates of BP control [38]. This was indeed the case in the DENERHTN trial where only 50% of the patients were fully adherent to multiple medications given in separate pills [39].

With respect to home BP, the reduction from baseline in home SBP was significantly greater in the renal denervation versus in the sham control group at 1 month post-procedure (−10.2 vs −4.8 mmHg, between-group difference −5.4 mmHg, *p* = 0.046). It is notable that in subjects without medication changes, this reduction was greater at 1 month post-procedure (−10.8 vs −3.6 mmHg, between-group difference −7.3 mmHg, *p* = 0.004), and the between-group difference was maintained at 2 months post-procedure (−9.4 vs −5.0 mmHg, between-group difference −4.4 mmHg, *p* = 0.050). This early reduction in BP might reflect the immediate effects of renal denervation on sympatholytic activity. However, because progressive reductions in home BP were also seen over time in the sham control group, between-group differences were not maintained at the 2- and 3-month follow-up. As discussed above, it is possible that the act of self-monitoring BP might have caused changes in patient behavior and/or unblinding which could have led to progressive reductions in BP during the study (irrespective of any other intervention), as has been described previously [40].

Another important factor is that patients with primary aldosteronism might not have been completely excluded from this study, even though this was one of the listed exclusion criteria, because 32.4% of patients showed hyperaldosteronism even when being treated with three or more antihypertensive drugs, including renin-angiotensin-aldosterone inhibitors.

### Study limitations

The inclusion of a sham control group is a key strength of this study, which is the first to evaluate the effects of ultrasound renal denervation in treated patients with resistant hypertension from Asia. In addition, this study focused on ambulatory BP as the primary outcome measure. Despite the neutral results, the data add to the current body of knowledge regarding the safety of renal denervation in patients with hypertension.

The study findings must be interpreted in light of several limitations. First, there was no standardization of antihypertensive medications or objective measurement of medication adherence using blood or urine. The lack of standardization in medications may have led to increased variability in BP outcomes. In addition, medication adherence is known to be a challenge in patients with resistant hypertension, especially when adherence is assessed by objective measures such as blood or urine metabolites [41]. Second, the nature of the intervention meant that it was not possible to conduct a double-blind study where medical personnel were unaware of treatment group allocation and, unlike other recent renal denervation studies, we did not prohibit unblinded physicians from participating in follow-up care. There was also no assessment of blinding conducted to determine whether or not the blinding was maintained. Third, there are significant seasonal variation of the temperature and BPs in Japan [42–47]. Morning BP increased in the winter, while the nighttime BP increase in the summer [46, 47].

## Conclusions and perspectives

Although BP decreased significantly from baseline in the denervation group, this trial had a neutral result because there was a similar reduction in BP in the sham control group. It is highly likely that this outcome reflects shortcomings in the design and conduct of this trial.

Our original approach in designing the REQUIRE trial followed naturalistic clinical practice principles. Patients with resistant hypertension were allowed to remain on their multi-drug pre-study treatment regimens, they were able to monitor their own treatment progress during the study by performing home BP measurements, and, consistent with the local traditions of close patient/physician relationships, research clinicians were not blinded to the randomized assignment of their patients to renal denervation or a sham procedure. Furthermore, in keeping with this approach, the timing of and adherence to medication taking were at the patients’ discretion (i.e., witnessed pill taking at the times of critical study observations and oversight by measurement of drug levels in blood and urine samples were not performed).

More positively, the lessons learned from this experience will enable us to now design a follow-up trial that will address the shortcomings identified in REQUIRE. This new trial should impose strict guidance on realistic drug regimens, and it needs to establish consistent timing of drug taking and witnessed pill-taking at critical stages of the study together with confirmation of treatment adherence by comprehensive blood and urine drug assays. In addition, the trial should maintain strict blinding of patients and physician observers to randomized treatment assignment. We believe that these rigorous steps, used successfully in the recent RADIANCE-HTN TRIO trial [16], will enable us to make a definitive evaluation of the safety and effectiveness of renal denervation in Asian patients with uncontrolled hypertension.

## Supplementary information

Supplemental Materials
