# Supplementary material for: Catheter-based ultrasound renal denervation in patients with resistant hypertension: the randomized, controlled REQUIRE trial
Source: Hypertens Res. 2021 Oct 15;45(2):221–31. doi: 10.1038/s41440-021-00754-7 (PMC8766280; doi:10.1038/s41440-021-00754-7)

**SUPPLEMENTAL MATERIAL**

**Catheter-based ultrasound renal denervation in patients with resistant hypertension: the randomized, controlled REQUIRE trial**

Kazuomi Kario^1^, Yoshiaki Yokoi^2^, Keisuke Okamura^3^, Masahiko Fujihara^2^, Yukako Ogoyama^1^, Eiichiro Yamamoto^4^, Hidenori Urata^3^, Jin-Man Cho^5^, Chong-Jin Kim^6^, Seung-Hyuk Choi^7^, Keisuke Shinohara^8^, Yasushi Mukai^9^, Tomokazu Ikemoto^10^, Masato Nakamura^11^, Shuichi Seki^12^, Satoaki Matoba^13^, Yoshisato Shibata^14^, Shigeo Sugawara^15^, Kazuhiko Yumoto^16^, Kouichi Tamura^17^, Fumiki Yoshihara^18^, Satoko Nakamura^19^, Woong Chol Kang^20^, Taro Shibasaki^21^, Keigo Dote^22^, Hiroyoshi Yokoi^23^, Akiko Matsuo^24^, Hiroshi Fujita^25^, Toshiyuki Takahashi^26^, Hyun-Jae Kang^27^, Yasushi Sakata^28^, Kazunori Horie^29^, Naoto Inoue^30^, Ken-ichiro Sasaki^31^, Takafumi Ueno^32^, Hirofumi Tomita^33^, Yoshihiro Morino^34^, Yuhei Nojima^35^, Chan Joon Kim^36^, Tomoaki Matsumoto^37^, Hisashi Kai^38^ and Shinsuke Nanto^39^

^1^Division of Cardiovascular Medicine, Department of Medicine, Jichi Medical University School of Medicine, Tochigi, Japan; ^2^Department of Cardiology, Kishiwada Tokushukai Hospital, Osaka, Japan; ^3^Department of Cardiovascular Diseases, Fukuoka University Chikushi Hospital, Fukuoka, Japan; ^4^Department of Cardiovascular Medicine, Kumamoto University Graduate School of Medical Science, Kumamoto, Japan; ^5^Division of Cardiology, Department of Internal Medicine, KyungHee University Hospital at Gangdong, Seoul, South Korea; ^6^Division of Cardiology, Department of Internal Medicine, CHA Gangnam Medical Center, Seoul, South Korea; ^7^Division of Cardiology Heart Vascular and Stroke Institute, Department of Medicine, Samsung Medical Center, Sungkyunkwan University School of Medicine, Seoul, South Korea; ^8^Department of Cardiovascular Medicine, Kyushu University Hospital, Fukuoka, Japan; ^9^Division of Cardiology, Fukuoka Red Cross Hospital, Fukuoka, Japan; ^10^Division of Cardiology, Kumamoto Red Cross Hospital, Kumamoto, Japan; ^11^Division of Cardiovascular Medicine, Toho University Ohashi Medical Center, Tokyo, Japan; ^12^Department of Cardiology, Chikamori Hospital, Kochi, Japan; ^13^Department of Cardiovascular Medicine, Graduate School of Medical Science, Kyoto Prefectural University of Medicine, Kyoto, Japan; ^14^Department of Cardiology, Miyazaki Medical Association Hospital, Miyazaki, Japan; ^15^Department of Cardiology, Nihonkai General Hospital, Yamagata, Japan; ^16^Department of Cardiology, Yokohama Rosai Hospital, Kanagawa, Japan; ^17^ Department of Medical Science and Cardiorenal Medicine Yokohama City University Medical Center, Kanagawa, Japan; ^18^Division of Nephrology and Hypertension, National Cerebral and Cardiovascular Center, Osaka, Japan; ^19^Department of Nutritional Science for Well-being, Kansai University of Welfare Sciences, Osaka, Japan; ^20^Department of Cardiology, Gil Medical Center, Gachon University College of Medicine, Incheon, South Korea; ^21^Department of Cardiology, Saitama Sekishinkai Hospital, Saitama, Japan; ^22^Department of Cardiology, Hiroshima City Asa Hospital, Hiroshima, Japan; ^23^Cardiovascular Center, Fukuoka Sanno Hospital, Fukuoka, Japan; ^24^Department of Cardiology, Japanese Red Cross Kyoto Daini Hospital, Kyoto, Japan; ^25^Department of Cardiology, North Medical Center, Kyoto Prefectural University of Medicine, Kyoto, Japan; ^26^Department of Cardiology, Saiseikai Central Hospital, Tokyo, Japan; ^27^Department of Internal Medicine, Seoul National University Hospital and University College of Medicine, Seoul National University, Seoul, South Korea; ^28^Department of Cardiovascular Medicine, Osaka University Graduate School of Medicine, Osaka, Japan; ^29^Department of Cardiovascular Medicine, Sendai Kousei Hospital, Miyagi, Japan; ^30^Cardiovascular Center, Tokyo Kamata Hospital, Tokyo, Japan; ^31^Division of Cardiovascular Medicine, Department of Internal Medicine, Kurume University School of Medicine, Fukuoka, Japan; ^32^Division of Cardiology, Fukuoka Kinen Hospital, Fukuoka, Japan; ^33^Department of Cardiology, Hirosaki University Graduate School of Medicine, Aomori, Japan; ^34^Division of Cardiology, Department of Internal Medicine, Iwate Medical University, Iwate, Japan; ^35^Department of Cardiovascular Medicine, Nishinomiya Municipal Central Hospital, Hyogo, Japan; ^36^Division of Cardiology, Department of Internal Medicine, Uijeongbu St Mary's Hospital, College of Medicine, The Catholic University of Korea, Seoul, South Korea; ^37^Department of Cardiology, Oji General Hospital, Hokkaido, Japan; ^38^Department of Cardiology, Kurume University Medical Center, Fukuoka, Japan; ^39^Department of Cardiovascular Medicine, Nishinomiya Municipal Central Hospital, Hyogo, Japan

**SUPPLEMENTARY METHODS**

**Study Sites and Principal Investigators**

| **Site** | **Principal investigator** |
| --- | --- |
| Division of Cardiology, Caress Sapporo Tokeidai Memorial Hospital, Hokkaido, Japan | Kazushi Urasawa |
| Division of Cardiology, Tonan Hospital, Hokkaido, Japan | Yutaka Matsui |
| Department of Cardiology, KKR Sapporo Medical Center Hospital, Hokkaido, Japan | Mitsunori Kamigaki |
| Department of Cardiology, Nayoro City General Hospital, Hokkaido, Japan | Masaru Yamaki |
| Department of Cardiology, Oji General Hospital, Hokkaido, Japan | Tomoaki Matsumoto |
| Department of Cardiology, Hirosaki University Graduate School of Medicine, Aomori, Japan | Hirofumi Tomita |
| Division of Cardiology, Department of Internal Medicine, Iwate Medical University, Iwate, Japan | Yoshihiro Morino |
| Department of Cardiovascular Medicine, Sendai Kousei Hospital, Miyagi, Japan | Naoto Inoue (Former Principal Investigator) |
|  | Kazunori Horie |
| Department of Cardiovascular Medicine, Tohoku University Graduate School of Medicine, Miyagi, Japan | Kei Takase |
| Department of Cardiology, Nihonkai General Hospital, Yamagata, Japan | Shigeo Sugawara |
| Division of Cardiovascular Medicine, Department of Medicine, Jichi Medical University School of Medicine, Tochigi, Japan | Kazuomi Kario |
| Department of Cardiology, Saitama Sekishinkai Hospital, Saitama, Japan | Taro Shibasaki |
| Department of Cardiology, Tokyo Bay Urayasu-Ichikawa Medical Center, Chiba, Japan | Tatsuya Nakama |
| Department of Cardiology, Nishiarai Heart Center Hospital, Tokyo, Japan | Katsumi Saito |
| Division of Cardiology, Mitsui Memorial Hospital, Tokyo, Japan | Jiro Aoki |
| Division of Cardiovascular Medicine, Toho University Ohashi Medical Center, Tokyo, Japan | Masato Nakamura |
| Department of Cardiology, Toranomon Hospital, Tokyo, Japan | Takahide Kodama |
| Department of Cardiology, Saiseikai Central Hospital, Tokyo, Japan | Toshiyuki Takahashi |
| Department of Cardiology, Minamino Cardiovascular Hospital, Tokyo, Japan | Yoshiki Hata |
| Department of Cardiology, Japanese Red Cross Musashino Hospital, Tokyo, Japan | Takashi Ashikaga |
| Department of Medical Science and Cardiorenal Medicine Yokohama City University Medical Center, Kanagawa, Japan | Kouichi Tamura |
| Department of Cardiology, Yokohama Rosai Hospital, Kanagawa, Japan | Kazuhiko Yumoto |
| Department of Cardiology, Yokohama Sakae Kyosai Hospital, Kanagawa, Japan | Ichiro Michishita |
| Department of Cardiology, Niigata City General Hospital, Niigata, Japan | Keiichi Tsuchida |
| Department of Cardiology, Kanazawa Medical University, Ishikawa, Japan | Michihiko Kitayama |
| Department of Cardiology, Kanazawa University Graduate School of Medicine, Ishikawa, Japan | Masayuki Takamura |
| Department of Cardiology, Chuno Kosei Hospital, Gifu, Japan | Tomohiko Iwata |
| Department of Cardiology, Suzuka Chuo General Hospital, Mie, Japan | Tetsuya Kitamura |
| Department of Cardiology, Japanese Red Cross Kyoto Daini Hospital, Kyoto, Japan | Hiroshi Fujita (Former Principal Investigator) |
|  | Akiko Matsuo |
| Department of Cardiovascular Medicine, Graduate School of Medical Science, Kyoto Prefectural University of Medicine, Kyoto, Japan | Satoaki Matoba |
| Cardiovascular Center, Kyoto Katsura Hospital, Kyoto, Japan | Shigeru Nakamura |
| Department of Cardiology, Kishiwada Tokushukai Hospital, Osaka, Japan | Yoshiaki Yokoi |
| Division of Cardiology, Osaka Rosai Hospital, Osaka, Japan | Masami Nishino |
| Division of Nephrology and Hypertension, National Cerebral and Cardiovascular Center, Osaka, Japan | Satoko Nakamura (Former Principal Investigator) |
|  | Fumiki Yoshihara |
| Department of Cardiovascular Medicine, Osaka University Graduate School of Medicine, Osaka, Japan | Yasushi Sakata |
| Division of Cardiovascular Medicine, Department of Internal Medicine, Kobe University Graduate School of Medicine, Hyogo, Japan | Toshiro Shinke (Former Principal Investigator) |
|  | Hidekazu Tanaka |
| Department of Cardiology, Cardiovascular Center, Kansai Rosai Hospital, Hyogo, Japan | Masaaki Uematsu (Former Principal Investigator) |
|  | Toshiaki Mano |
| Department of Cardiovascular Medicine, Nishinomiya Municipal Central Hospital, Hyogo, Japan | Yuhei Nojima |
| Department of Cardiology, The Sakakibara Heart Institute of Okayama, Okayama, Japan | Atushi Hirohata |
| Department of Cardiology Okayama Rosai Hospital, Okayama, Japan | Seiji Namba |
| Department of Cardiology, Hiroshima City Asa Hospital, Hiroshima, Japan | Keigo Dote |
| Department of Cardiology, Pulmonology, Hypertension and Nephrology, Ehime University Graduate School of Medicine, Ehime, Japan | Takafumi Okura (Former Principal Investigator) |
|  | Osamu Yamaguchi |
| Department of Cardiology, Chikamori Hospital, Kochi, Japan | Shuichi Seki |
| Department of Cardiovascular Medicine, Kyushu University Hospital, Fukuoka, Japan | Yasushi Mukai (Former Principal Investigator) |
|  | Keisuke Shinohara |
| Cardiovascular Center, Fukuoka Sanno Hospital, Fukuoka, Japan | Hiroyoshi Yokoi |
| Department of Cardiovascular Diseases, Fukuoka University Chikushi Hospital, Fukuoka, Japan | Hidenori Urata |
| Department of Cardiovascular Medicine, Fukuoka Tokushukai Medical Center, Fukuoka, Japan | Hideki Shimomura |
| Department of Cardiology, Kokura Memorial Hospital, Fukuoka, Japan | Kenji Ando |
| Division of Cardiovascular Medicine, Department of Internal Medicine, Kurume University School of Medicine, Fukuoka, Japan | Takafumi Ueno (Former Principal Investigator) |
|  | Ken-ichiro Sasaki |
| Department of Cardiovascular Medicine, Saga University, Saga, Japan | Yutaka Hikichi (Former Principal Investigator) |
|  | Machiko Asaka |
| Department of Cardiovascular Medicine, Kumamoto University Graduate School of Medical Science, Kumamoto, Japan | Eiichiro Yamamoto |
| Division of Cardiology, Kumamoto Red Cross Hospital, Kumamoto, Japan | Tomokazu Ikemoto |
| Department of Cardiology, Miyazaki Medical Association Hospital, Miyazaki, Japan | Yoshisato Shibata |
| Department of Cardiovascular Medicine and Hypertension, Graduate School of Medical and Dental Sciences, Kagoshima University, Kagoshima, Japan | Mitsuru Ohishi |
| Department of Cardiovascular Medicine, Nephrology and Neurology, University of the Ryukyus, Okinawa, Japan | Hidekazu Ikemiyagi |
| Division of Cardiology, Department of Internal Medicine, KyungHee University Hospital at Gangdong, Seoul, South Korea. | Chong-Jin Kim (Former Principal Investigator) |
|  | Jin-Man Cho |
| Division of Cardiology, Department of Internal Medicine, Gangnam Severance Hospital, Yonsei University College of Medicine, Seoul, South Korea. | Pil-Ki Min |
| Division of Cardiology Heart Vascular and Stroke Institute, Department of Medicine, Samsung Medical Center, Sungkyunkwan University School of Medicine, Seoul, South Korea. | Seung-Hyuk Choi |
| Department of Internal Medicine, Cardiovascular Center, Guro Hospital, Korea University, Seoul, South Korea. | Chang-gyu Park |
| Department of Internal Medicine, Seoul National University Hospital and  University College of Medicine, Seoul National University, Seoul, South Korea. | Hyun-Jae Kang |
| Division of Cardiology, Department of Internal Medicine, Seoul St. Mary's Hospital, College of Medicine, The Catholic University of Korea, Seoul, South Korea. | Woo-Baek Chung |
| Division of Cardiology, Department of Internal Medicine, Severance Cardiovascular Hospital, Yonsei University Health System, Yonsei University College of Medicine, Seoul, South Korea. | Byeong-Keuk Kim |
| Division of Cardiology, Department of Internal Medicine, Cardiovascular Center, Seoul National University Bundang Hospital, Seongnam, South Korea. | Cheol-Ho Kim |
| Division of Cardiology, Department of Internal Medicine, Uijeongbu St Mary's Hospital, College of Medicine, The Catholic University of Korea, Seoul, South Korea. | Chan Joon Kim |
| Department of Cardiology, Gil Medical Center, Gachon University College of Medicine, Incheon, South Korea. | Woong Chol Kang |
| Department of Cardiology, Gangneung Asan Hospital, University of Ulsan College of Medicine, Gangneung, South Korea. | Sang-Yong Yoo |
| Division of Cardiology, Department of Internal Medicine, Wonju Severance Christian Hospital, Yonsei University Wonju College of Medicine, Wonju, South Korea. | Sung-Gyun Ahn |
| Division of Cardiology, Department of Internal Medicine, Chungnam National University Hospital, Chungnam National University College of Medicine, Daejeon, South Korea. | Jin-Ok Jeong |
| Division of Cardiology, Department of Internal Medicine, Chonnam National University Hospital, Chonnam National University Medical School, Gwangju, South Korea. | Young-keun Ahn |
| Division of Cardiology, Department of Internal Medicine, Pusan National University Hospital, Busan, South Korea. | Han-Cheol Lee |
| Division of Cardiology, Department of Internal Medicine, Keimyung University Dongsan Hospital, Daegu, South Korea. | Seung-Ho Hur |
| Division of Cardiology, Department of Internal Medicine, Pusan National University Yangsan Hospital, Yangsan, South Korea. | Kook-Jin Chun |

**Administrative Structure**

| **Role and Name** | **Institute** |
| --- | --- |
| **Coordinating Investigators:** |  |
| Shinsuke Nanto, MD, PhD | Department of Cardiovascular Medicine, Nishinomiya Municipal Central Hospital, Hyogo, Japan |
| Kazuomi Kario, MD, PhD | Division of Cardiovascular Medicine, Department of Medicine, Jichi Medical University School of Medicine, Tochigi, Japan |
| **Medical Experts:** |  |
| Hisahi Kai, MD, PhD | Department of Cardiology, Kurume University Medical Center, Fukuoka, Japan |
| Satoshi Hoshide, MD, PhD | Division of Cardiovascular Medicine, Department of Medicine, Jichi Medical University School of Medicine, Tochigi, Japan |
| **Advisor on Renal Artery Access:** |  |
| Yoshiaki Yokoi, MD, PhD | Department of Cardiology, Kishiwada Tokushukai Hospital, Osaka, Japan |
| **Advisory Team for Ablation Sites:** |  |
| Shinsuke Nanto, MD, PhD | Department of Cardiovascular Medicine, Nishinomiya Municipal Central Hospital, Hyogo, Japan |
| Ryoji Koshida, MD | Department of Cardiology, Toyohashi Heart Center, Toyohashi, Aichi, Japan |
| Keisuke Okamura, MD, PhD | Department of Cardiovascular Diseases, Fukuoka University Chikushi Hospital, Fukuoka, Japan |
| Masahiko Fujihara, MD | Department of Cardiology, Kishiwada Tokushukai Hospital, Osaka, Japan |
| Yukako Ogoyama, MD | Division of Cardiovascular Medicine, Department of Medicine, Jichi Medical University School of Medicine, Tochigi, Japan |

**SUPPLEMENTARY TABLES**

**Supplementary table 1.** Procedural details

|  | **Renal denervation**  **(n=72)** | **Sham control**  **(n=71)** |
| --- | --- | --- |
| Proportion of patients with ≥2 sonication on each renal artery | 71 (98.6) | - |
| Total number of sonications at both arteries | 5.5 ± 1.2 | - |
| Subjects with treated accessory renal arteries | 8 (11.1) | - |
| Procedure time, min* | 86.7 ± 54.0 | 40.2 ± 11.6 |
| Renal denervation procedure time, min^†^ | 35.5 ± 23.8 | - |
| Contrast volume, mL | 147.8 ± 85.8 | 54.1 ± 36.5 |
| X-ray fluoroscopy time, min | 23.6 ± 12.8 | 5.2 ± 5.1 |

Mean ± SD, or number of patients (%)

*Time from sheath insertion to removal.

^†^Time from first Paradise™ catheter insertion to the last removal of the Paradise™ catheter.

**Supplementary table 2.** Change from baseline in blood pressure between the renal denervation and sham control groups at 3 months

| **Variables** | **Renal denervation**  **(mmHg)** | | **Sham control**  **(mmHg)** | | **Between-group difference (mmHg)** | |
| --- | --- | --- | --- | --- | --- | --- |
|  | **N** | **LS mean** ± **SE** | **n** | **LS mean** ± **SE** | **LS mean** ± **SE** | **p-value** |
| Office SBP | 69 | –11.0 ± 2.1 | 66 | –9.0 ± 2.1 | –2.0 ± 3.0 | 0.511 |
| Office DBP | 69 | –4.9 ± 1.5 | 66 | –5.0 ± 1.5 | 0.1 ± 2.1 | 0.946 |
| 24-hour ambulatory SBP | 69 | –6.6 ± 1.9 | 67 | –6.5 ± 1.9 | –0.1 ± 2.7 | 0.971 |
| 24-hour ambulatory DBP | 69 | –3.6 ± 1.0 | 67 | –3.3 ± 1.0 | –0.4 ± 1.4 | 0.806 |
| Daytime ambulatory SBP | 61 | –8.4 ± 2.0 | 66 | –7.2 ± 1.9 | –1.2 ± 2.8 | 0.672 |
| Daytime ambulatory DBP | 61 | –4.8 ± 1.1 | 66 | –4.0 ± 1.0 | –0.8 ± 1.5 | 0.585 |
| Nighttime ambulatory SBP | 68 | –4.2 ± 2.4 | 67 | –4.7 ± 2.4 | 0.5 ± 3.3 | 0.883 |
| Nighttime ambulatory DBP | 68 | –1.4 ± 1.3 | 67 | –2.0 ± 1.3 | 0.6 ± 1.9 | 0.770 |
| Home SBP | 60 | –8.7 ± 1.8 | 59 | –6.9 ± 1.8 | –1.8 ± 2.6 | 0.488 |
| Home DBP | 60 | –3.6 ± 1.1 | 59 | –3.7 ± 1.1 | 0.1 ± 1.6 | 0.949 |
| Morning home SBP | 60 | –9.1 ± 1.8 | 59 | –6.6 ± 1.8 | –2.5 ± 2.5 | 0.319 |
| Morning home DBP | 60 | –3.7 ± 1.2 | 59 | –3.1 ± 1.2 | –0.7 ± 1.7 | 0.684 |

ANCOVA model included procedure as a factor and baseline value as a covariate.

DBP, diastolic blood pressure; LS, least squares; SBP, systolic blood pressure; SE, standard error.

**Supplementary table 3.** Medication changes

| **Period** | **Renal denervation** | | | | **Sham control** | | | |
| --- | --- | --- | --- | --- | --- | --- | --- | --- |
|  | **Number of antihypertensive medications** | | **Antihypertensive load index*** | | **Number of antihypertensive medications** | | **Antihypertensive load index*** | |
|  | **N** | **Mean ± SD** | **N** | **Mean ± SD** | **N** | **Mean ± SD** | **N** | **Mean ± SD** |
| Informed consent | 69 | 4.2 ± 1.6 | - | - | 67 | 4.0 ± 1.1 | - | - |
| Baseline | 62 | 4.2 ± 1.7 | 69 | 2.6 ± 1.7 | 62 | 3.9 ± 1.2 | 67 | 2.4 ± 1.2 |
| 1 month | 59 | 4.1 ± 1.6 | - | - | 57 | 3.9 ± 1.2 | - | - |
| 2 month | 60 | 4.2 ± 1.6 | - | - | 60 | 3.9 ± 1.1 | - | - |
| 3 month | 60 | 4.3 ± 1.7 | 69 | 2.5 ± 1.7 | 59 | 3.9 ± 1.1 | 67 | 2.4 ± 1.2 |

*Antihypertensive load index is the sum of (daily dose/maximum daily dose) for each antihypertensive drug.

SD, standard deviation.

**Table S4.** Change from baseline in blood pressure between the renal denervation and sham control groups at 3 months in patients without any change of antihypertensive drugs

| **Variables** | **Renal denervation**  **(mmHg)** | | **Sham control**  **(mmHg)** | | **Between-group difference (mmHg)** | |
| --- | --- | --- | --- | --- | --- | --- |
|  | **N** | **LSmean** ± **SE** | **n** | **LSmean** ± **SE** | **LSmean** ± **SE** | **p-value** |
| Office SBP | 60 | –10.5 ± 2.1 | 60 | –7.8 ± 2.1 | –2.7 ± 2.9 | 0.370 |
| Office DBP | 60 | –4.3 ± 1.5 | 60 | –3.8 ± 1.5 | –0.5 ± 2.1 | 0.795 |
| 24-hour ambulatory SBP | 60 | –6.2 ± 1.9 | 61 | –5.7 ± 1.9 | –0.5 ± 2.7 | 0.844 |
| 24-hour ambulatory DBP | 60 | –3.2 ± 1.0 | 61 | –2.6 ± 1.0 | –0.6 ± 1.5 | 0.699 |
| Daytime ambulatory SBP | 53 | –7.8 ± 2.0 | 60 | –6.6 ± 1.9 | –1.2 ± 2.7 | 0.667 |
| Daytime ambulatory DBP | 53 | –4.4 ± 1.1 | 60 | –3.5 ± 1.0 | –0.9 ± 1.5 | 0.537 |
| Nighttime ambulatory SBP | 59 | –3.3 ± 2.5 | 61 | –3.5 ± 2.5 | 0.2 ± 3.6 | 0.952 |
| Nighttime ambulatory DBP | 59 | –0.8 ± 1.4 | 61 | –1.2 ± 1.4 | 0.4 ± 2.0 | 0.852 |
| Home SBP | 53 | –8.3 ± 1.8 | 55 | –5.9 ± 1.8 | –2.4 ± 2.5 | 0.347 |
| Home DBP | 53 | –3.6 ± 1.1 | 55 | –3.3 ± 1.1 | –0.4 ± 1.6 | 0.816 |
| Morning home SBP | 53 | –8.5 ± 1.8 | 55 | –5.8 ± 1.7 | –2.8 ± 2.5 | 0.268 |
| Morning home DBP | 53 | –3.8 ± 1.2 | 55 | –2.7 ± 1.2 | –1.1 ± 1.7 | 0.518 |

ANCOVA model included procedure as a factor and baseline value as a covariate.

DBP, diastolic blood pressure; LS, least squares; SBP, systolic blood pressure; SE, standard error.

**SUPPLEMENTARY FIGURES**

**Supplementary figure 1.** Between-group differences in 24-hour ambulatory systolic blood pressure in patient subgroups. The ANCOVA model included procedure and baseline value. The interaction p-value was calculated using an ANCOVA model including procedure, baseline value, baseline characteristics, and interaction between procedure and baseline characteristics. CI, confidence interval.

**
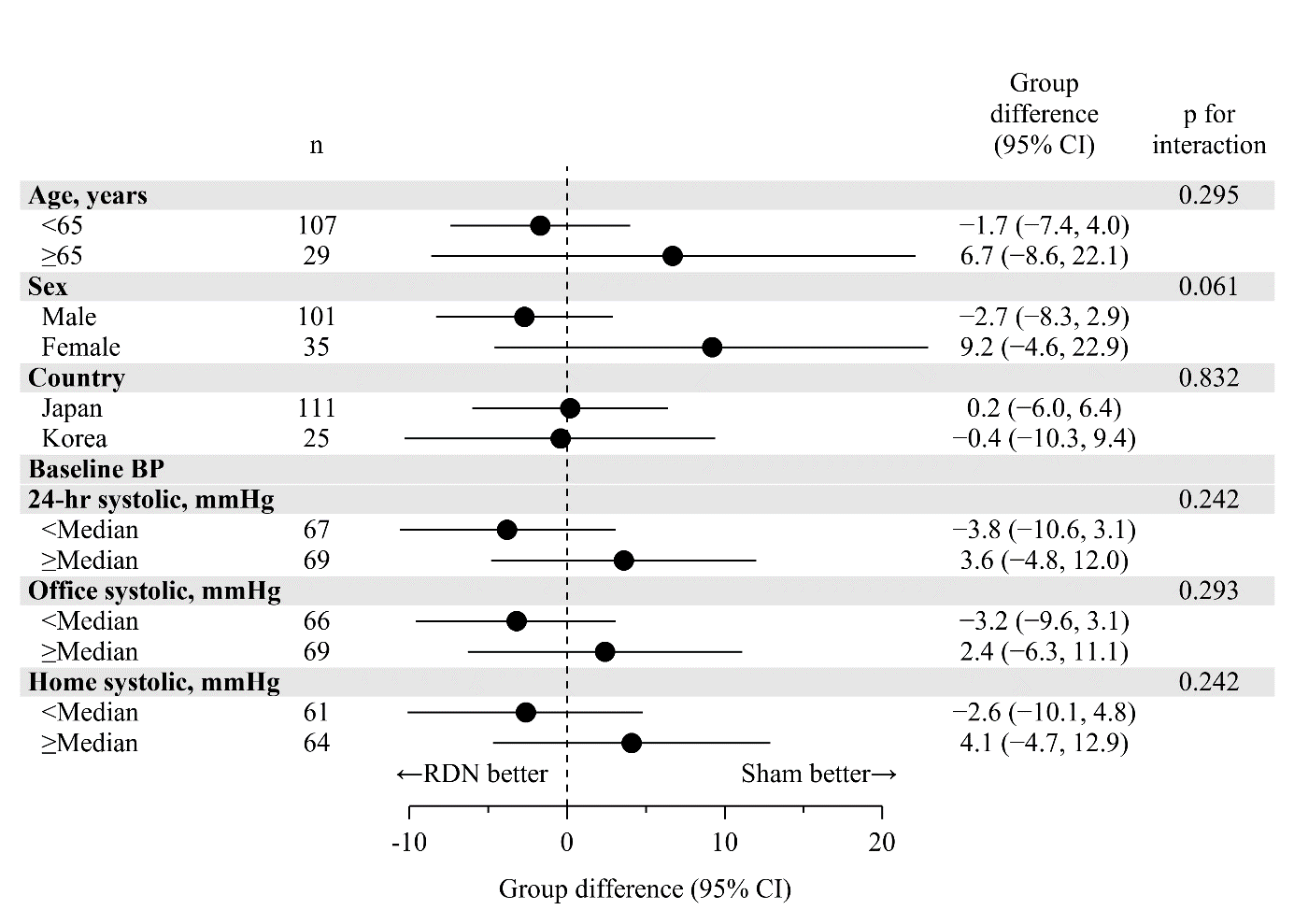
**

**Supplementary figure 2.** Individual patient changes in twenty-four-hour ambulatory systolic blood pressure (ABP) from baseline at 3 months post-procedure in the renal denervation (**A**) and sham control (**B**) groups. ABP, ambulatory blood pressure.

**A. Renal denervation group**


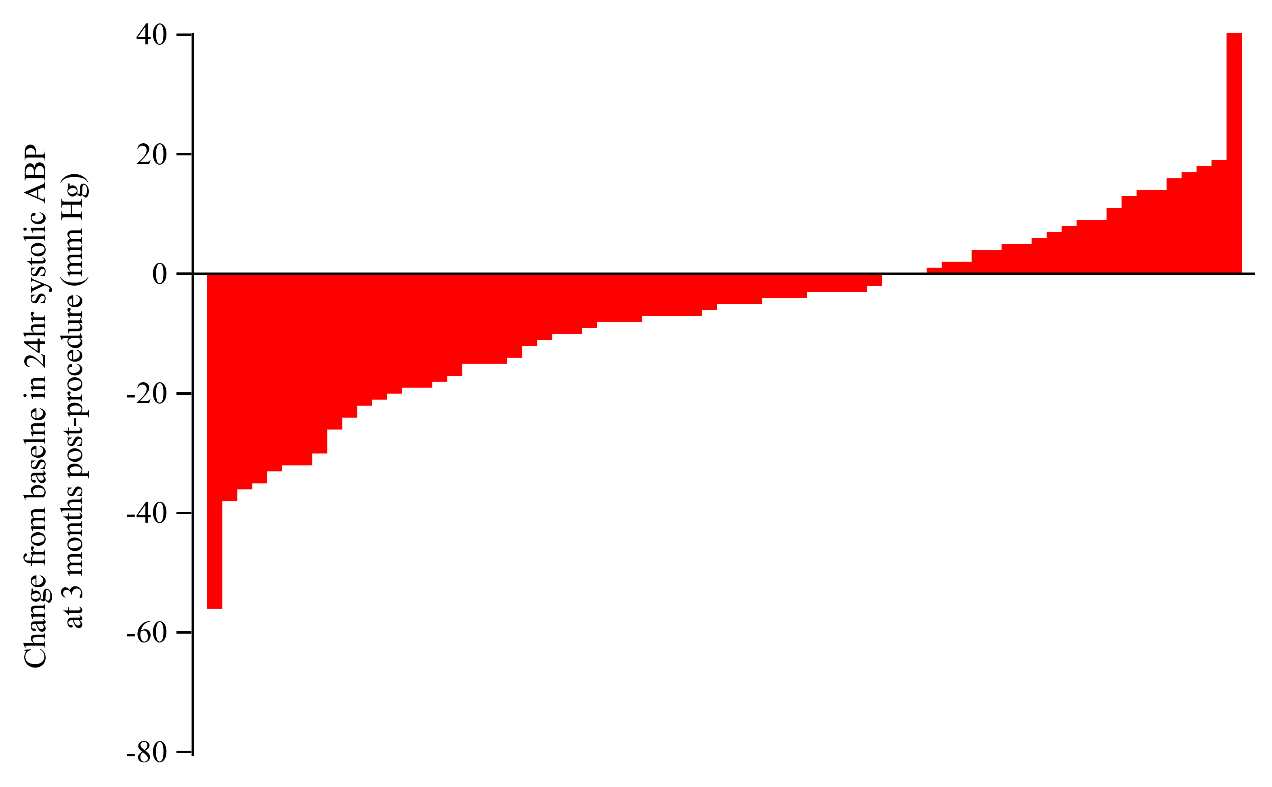


**B. Sham control group**


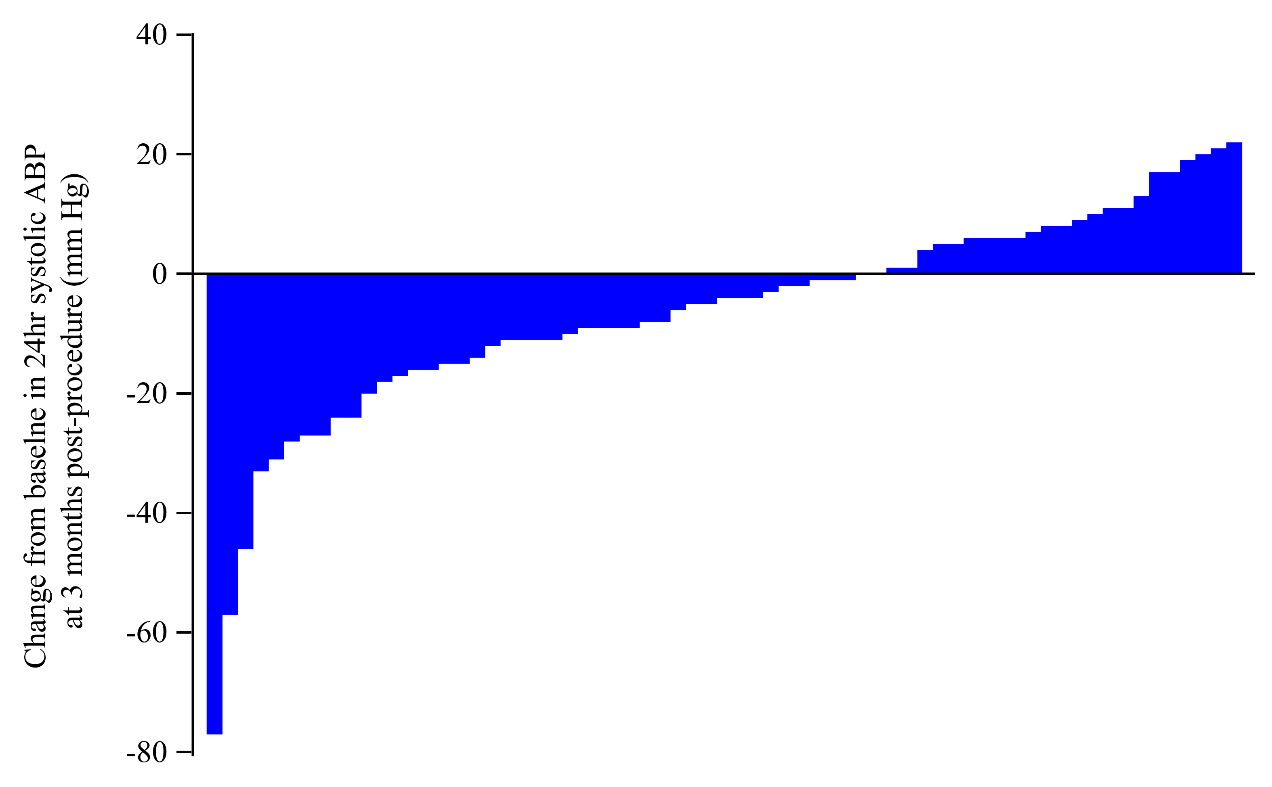


**Supplementary figure 3.** Twenty-four-hour ambulatory blood pressure profiles at baseline (empty dot) and at 3 months post-procedure (filled dot) in the renal denervation (**A**) and sham control (**B**) groups. Values are mean (dots) and standard error (SE; bars).

**A. Renal denervation group**


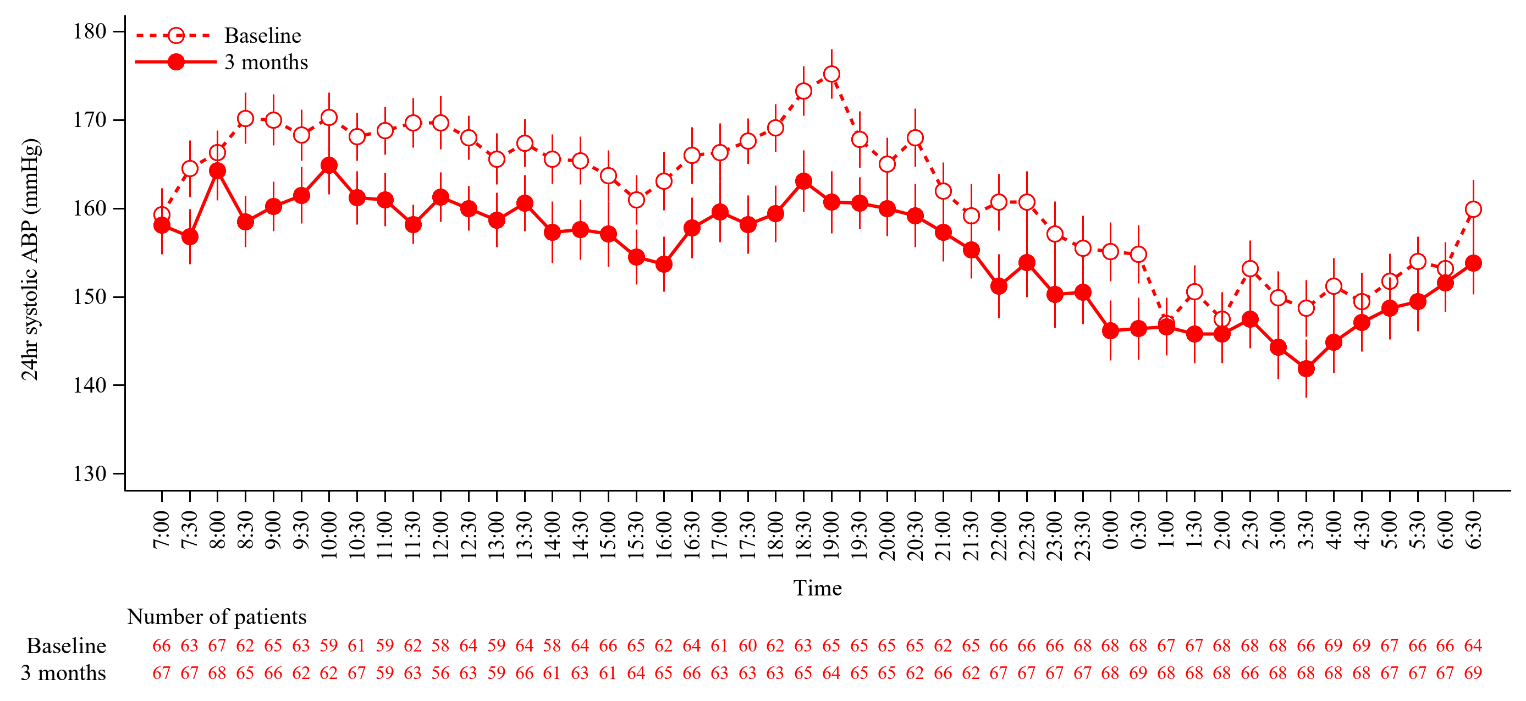


**B. Sham control group**


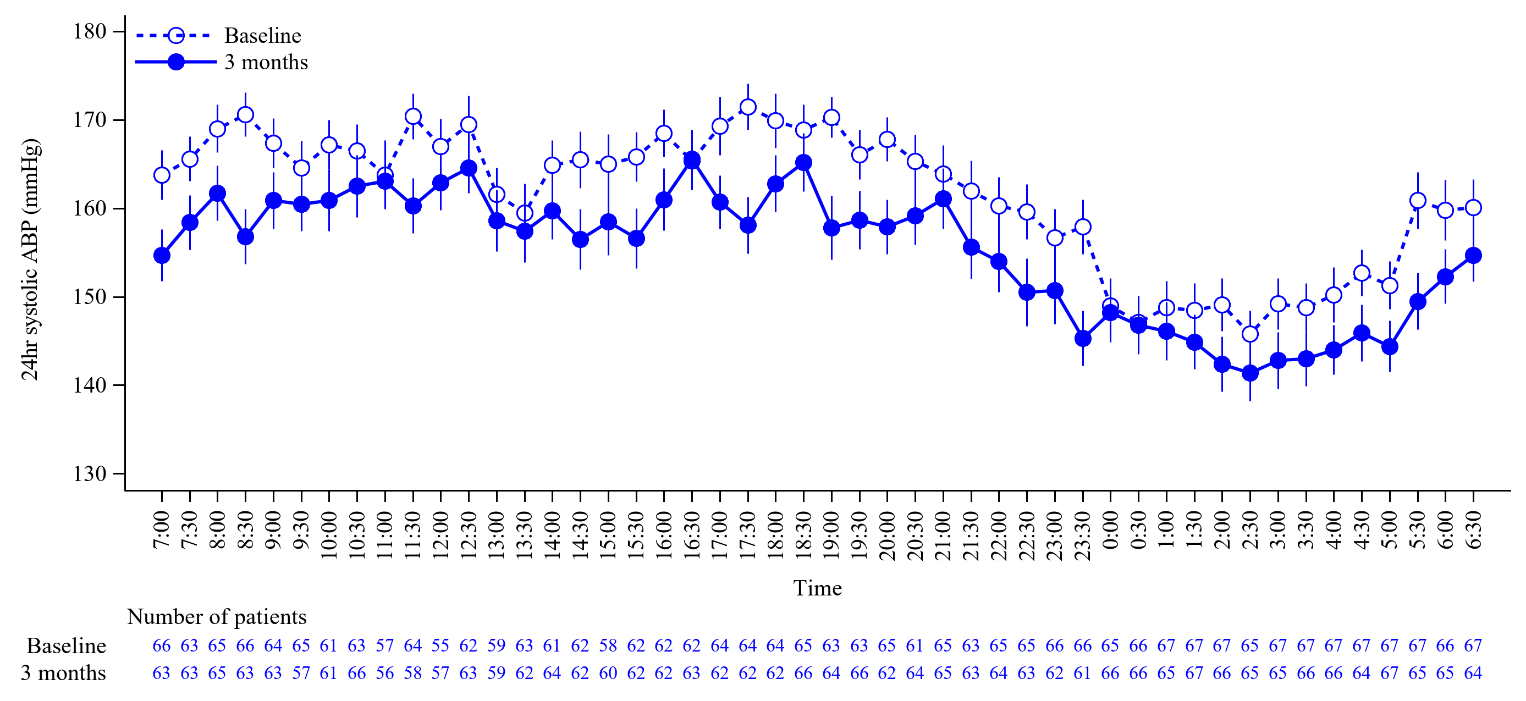


**Supplementary figure 4.** Change in home systolic blood pressure (SBP) over time after the procedure in patients without any change of antihypertensive drugs. Dots and error bars show least squares mean ± standard errors.


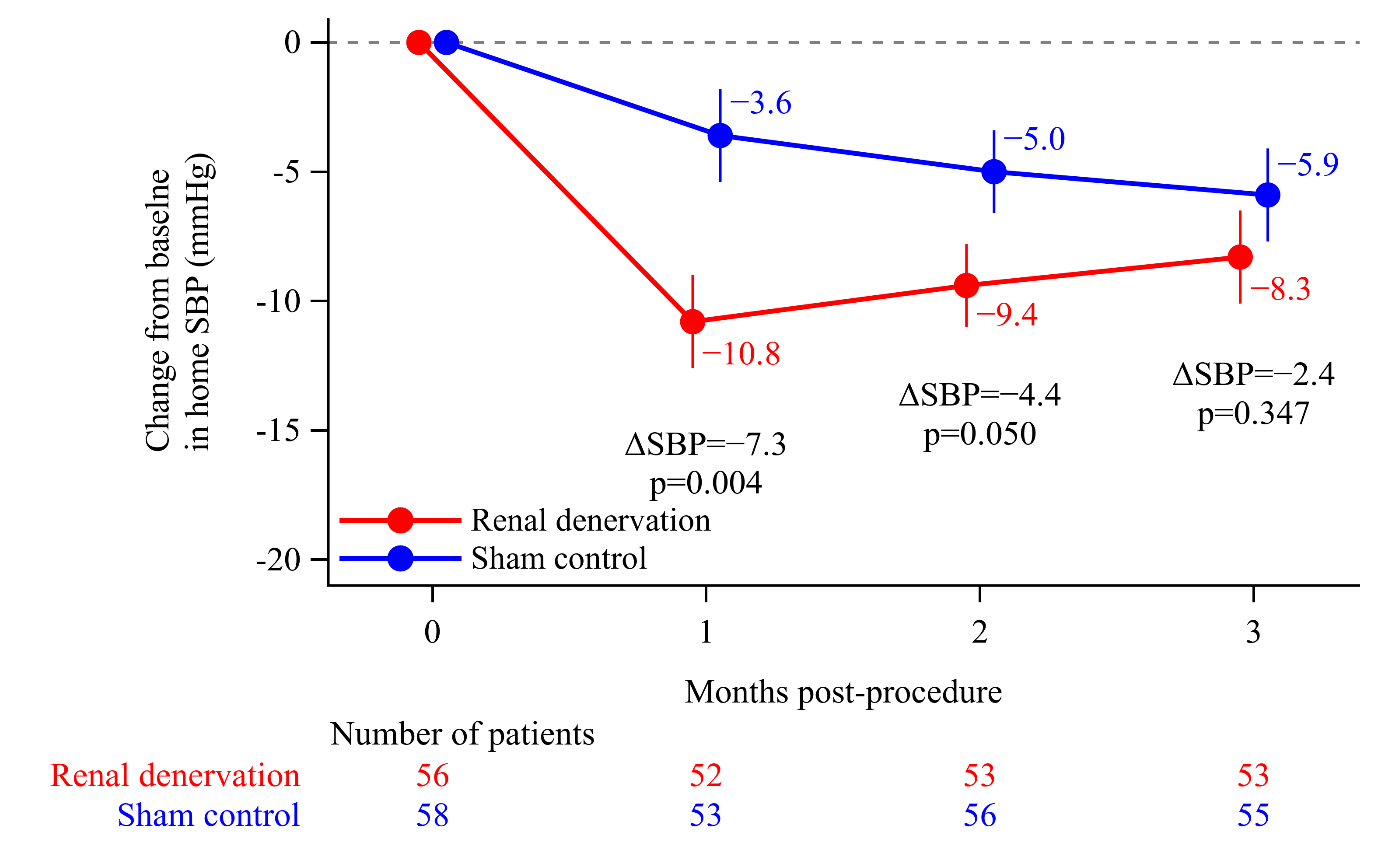

Supplement: Supplementary file 1 — Supplemental Materials [file 41440_2021_754_MOESM1_ESM.docx]
